# Establishing reference intervals for triglyceride-containing lipoprotein subfraction metabolites measured using nuclear magnetic resonance spectroscopy in a UK population

**DOI:** 10.1177/0004563220961753

**Published:** 2020-10-21

**Authors:** Roshni Joshi, Goya Wannamethee, Jorgen Engmann, Tom Gaunt, Deborah A Lawlor, Jackie Price, Olia Papacosta, Tina Shah, Therese Tillin, Peter Whincup, Nishi Chaturvedi, Mika Kivimaki, Diana Kuh, Meena Kumari, Alun D Hughes, Juan P Casas, Steve E Humphries, Aroon D Hingorani, A Floriaan Schmidt, on behalf of the UCLEB Consortium

**Affiliations:** 1 Institute of Cardiovascular Science, Faculty of Population Health, University College London, London, UK; 2 Department of Primary Care & Population Health, Faculty of Population Health, University College London, London, UK; 3 MRC Integrative Epidemiology Unit at the University of Bristol, Bristol, UK; 4 Bristol NIHR Biomedical Research Centre, Bristol, UK; 5 Population Health Science, Bristol Medical School, Bristol, UK; 6 Usher Institute, University of Edinburgh, Edinburgh, UK; 7 Department of Epidemiology and Public Health, University College London, London, UK; 8 Population Health Research Institute, St George’s, University of London, London, UK; 9 MRC Unit for Lifelong Health and Ageing, Institute of Cardiovascular Science, Faculty of Population Health Sciences, University College London, London, UK; 10 Institute for Social and Economic Research, University of Essex, Colchester, UK; 11 Massachusetts Veterans Epidemiology Research and Information Center (MAVERIC), VA Boston Healthcare, MA, USA; 12 Division of Aging, Department of Medicine, Brigham and Women’s Hospital and Harvard School of Medicine, Boston, MA, USA; 13 Department of Cardiology, Division Heart and Lungs, University Medical Center Utrecht, Utrecht, the Netherlands

**Keywords:** Analytes, clinical studies, epidemiology studies, laboratory methods, lipids, nuclear magnetic resonance

## Abstract

**Background:**

Nuclear magnetic resonance (NMR) spectroscopy allows triglycerides to be subclassified into 14 different classes based on particle size and lipid content. We recently showed that these subfractions have differential associations with cardiovascular disease events. Here we report the distributions and define reference interval ranges for 14 triglyceride-containing lipoprotein subfraction metabolites.

**Methods:**

Lipoprotein subfractions using the Nightingale NMR platform were measured in 9073 participants from four cohort studies contributing to the UCL-Edinburgh-Bristol consortium. The distribution of each metabolite was assessed, and reference interval ranges were calculated for a disease-free population, by sex and age group (<55, 55–65, >65 years), and in a subgroup population of participants with cardiovascular disease or type 2 diabetes. We also determined the distribution across body mass index and smoking status.

**Results:**

The largest reference interval range was observed in the medium very-low density lipoprotein subclass (2.5th 97.5th percentile; 0.08 to 0.68 mmol/L). The reference intervals were comparable among male and female participants, with the exception of triglyceride in high-density lipoprotein. Triglyceride subfraction concentrations in very-low density lipoprotein, intermediate-density lipoprotein, low-density lipoprotein and high-density lipoprotein subclasses increased with increasing age and increasing body mass index. Triglyceride subfraction concentrations were significantly higher in ever smokers compared to never smokers, among those with clinical chemistry measured total triglyceride greater than 1.7 mmol/L, and in those with cardiovascular disease, and type 2 diabetes as compared to disease-free subjects.

**Conclusion:**

This is the first study to establish reference interval ranges for 14 triglyceride-containing lipoprotein subfractions in samples from the general population measured using the nuclear magnetic resonance platform. The utility of nuclear magnetic resonance lipid measures may lead to greater insights for the role of triglyceride in cardiovascular disease, emphasizing the importance of appropriate reference interval ranges for future clinical decision making.

## Introduction

Risk factors for atherosclerotic disease include elevated total cholesterol, LDL-cholesterol (LDL-C) and triglycerides (TGs), and are used in disease risk assessment in clinical care.^1^ Elevated TGs are common among people with metabolic syndrome, obesity and type 2 diabetes (T2DM),^2^,^3^ and are associated with an increased risk of cardiovascular disease (CVD).^4^ Population-based reference intervals are used as a tool to define thresholds for clinical decisions. For example, clinical measurement of LDL-C for CVD risk assessment, which together with other measurements such as body mass index (BMI) or systolic blood pressure (SBP) can help to determine if lipid-lowering is indicated.^5^

The high-throughput proton (^1^H) serum nuclear magnetic resonance (NMR) metabolomics platform developed by Nightingale provides quantitative information on lipoprotein particle size and lipid content representing multiple metabolic pathways.^6–8^ NMR measures of lipoproteins are increasingly used in epidemiological and genetic studies, and may provide better insights into biological processes when compared to clinical chemistry measures of TG, which represent the sum of all plasma TG.^9^

The Nightingale NMR metabolomics approach has been used in two recent prospective cohort studies that found evidence to suggest a differential association of total serum TG and TG subfractions with coronary heart disease (CHD).^10^,^11^ For example, total serum TG association with CHD was OR 1.19 (95% CI 1.10 to 1.28), TG in the VLDL subfractions was associated with CHD in the range of OR 1.12 to 1.22, whereas TG in the LDL sub-fractions conveyed a relatively lower risk (OR in the range 1.13 to 1.17).^11^ The different associations of the 14 TG-subfractions with CHD highlight the need to extend the standard lipid reference intervals to include lipid lipoprotein subfractions.

In the present study, we aim to define reference intervals for 14 TG-containing lipoprotein subfraction metabolites (TG subfractions) using data from multiple UK-based cohorts from the UCL-Edinburgh-Bristol (UCLEB) consortium.

## Methods

### Population study sample

We sourced data from the UCLEB consortium, including NMR metabolite measures in 9073 participants from four cohort studies: The British Regional Heart Study (BRHS), including male aged 60–79 at assessment in 1998–2000, the Whitehall II Study (WHII), including UK government workers aged 45–69 years at assessment in 1997 to 1999, the Southall and Brent Revisited Study (SABRE), a tri-ethnic study including British men and women from European (SABRE1), South Asian (SABRE2) and African Caribbean (SABRE3) descent, and the Caerphilly Prospective Study (CAPS), including male registered in general practice aged 55–69 at assessment in 1989–1993. The design and data collection for the UCLEB Consortium of longitudinal population studies have been described previously.^12^ Age (years), sex (male/female), smoking (ever/never), BMI, CHD, stroke and T2DM variables were collected at the time of NMR blood sample measurement.

### Metabolite quantification

Using Nightingale NMR metabolomics platform,^8^ high-throughput metabolite quantification of 14 TG-containing lipoprotein subfractions (mmol/L) was ascertained in fasting and non-fasting serum samples in all contributing studies. To ensure long-term sample integrity, blood samples were stored and transported at −80°C across all contributing UCLEB studies until NMR quantification in 2014. NMR metabolomics platform has been extensively used in epidemiology and genetics studies,^13–15^ and its application reviewed and described in detail elsewhere.^6^,^16^

### Statistical analysis

We removed individuals based on any event of CHD, stroke or T2DM to include a healthy, ‘disease free’ population. We first assessed the study-specific distribution of each 14 TG subfraction and then pooled individual participant data form all four cohorts into one dataset. Reference intervals were based on the 2.5th and 97.5th percentiles stratified by age and sex. Age group bands were calculated as <55 years, 55–65 years and >65 years. The influence of fasting, age, smoking and BMI on the subfraction distributions was assessed statistically and graphically using ‘generalised linear model’ (GAM) curves, Kolmogorov–Smirnov (KS) test and using box plots. Reference intervals were additionally calculated in the following groups; (1) participants with CVD (defined as occurrence of either CHD or stroke), (2) participants with T2DM, (3) participants with clinical chemistry total TG greater, or less than 1.7 mmol/L and (4) TG measured in the fasting and non-fasting state.

## Results

A total of 9073 individuals were included in the main healthy, free of CVD and T2DM study sample, of which 5574 (62.8%) were male (median age 61.7 years, IQR 52.0, 67.6) who had median BMI of 26.0 (IQR 23.9, 28.4) kg/m^2^ and 3027 (54.2%) were current or ex-smokers (i.e. ever smokers). Female participants had a median age of 53.9 (IQR 49.9, 59.9), a BMI of 25.6 (23.6, 27.8) kg/m^2^ and 431 (13.0%) were ever smokers. Description of study population and median concentration of 14 subfractions is shown in Table 1.

**Table 1.** Description of study sample.

	Male (*n* = 5574)	Female (*n* = 3299)
Age, years	61.7 (52.0, 67.6)	53.9 (49.9, 59.9)
BMI, kg/m^2^	26.0 (23.9, 28.4)	25.6 (23.6, 27.8)
Smoking, ever	3027/5574 (54.2)	431/3299 (13.0)
Triglyceride subfraction (mmol/L)		
Extremely large VLDL	0.02 (0.01–0.03)	0.02 (0.01–0.02)
Very large VLDL	0.03 (0.01–0.05)	0.02 (0.01–0.04)
Large VLDL	0.10 (0.06–0.18)	0.09 (0.05–0.16)
Medium VLDL	0.23 (0.16–0.35)	0.24 (0.16–0.34)
Small VLDL	0.22 (0.18–0.29)	0.23 (0.17–0.30)
Very small VLDL	0.11 (0.09–0.13)	0.12 (0.09–0.14)
IDL	0.12 (0.10–0.14)	0.12 (0.10–0.15)
Large LDL	0.10 (0.08–0.12)	0.10 (0.09–0.12)
Medium LDL	0.05 (0.04–0.06)	0.05 (0.04–0.05)
Small LDL	0.03 (0.02–0.04)	0.03 (0.02–0.04)
Very large HDL	0.01 (0.01–0.02)	0.01 (0.01–0.02)
Large HDL	0.03 (0.02–0.04)	0.02 (0.02–0.03)
Medium HDL	0.05 (0.04–0.06)	0.05 (0.04–0.06)
Small HDL	0.05 (0.04–0.06)	0.04 (0.04–0.05)

Note: Values are median (IQR) or %.

VLDL: very-low density lipoprotein; IDL: intermediate-density lipoprotein; LDL: low-density lipoprotein; HDL: high-density lipoprotein

We compared the sum of TG in the 14 subfractions to clinical chemistry measured total TG and found an increase of 0.34 mmol/L of NMR measured total TG for every 1 mmol/L increase in clinical chemistry measured total TG (Supplemental Figure 1). The overall study population distribution for the 14 TG subfractions was comparable across contributing studies, showed agreement between ethnicities in the Southall and Brent Revisited Study cohort (Supplemental Figure 2) and an overlap of TG measured in the fasting and non-fasting state (Supplemental Figure 3). Of the 14 TG subfractions, 12 had a skewed right tailed distribution, and two (medium and small HDL) had a more symmetrical distribution.

**Figure 1.**
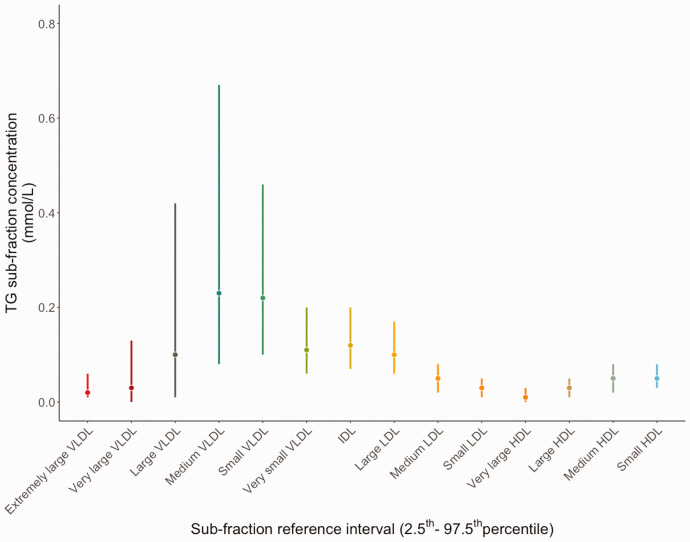
Reference interval range for 14 triglyceride subfractions (median, 2.5th, 97.5th) percentiles.Note. Please refer to the online version of the article to view the figure in colour.

The reference intervals (2.5th–97.5th) percentile for 14 TG subfractions are shown in Table 2 and graphically in Figure 1. Wide reference intervals were observed in the VLDL subclass, for example the reference interval for TG in medium VLDL and small VLDL was 0.08–0.67 mmol/L and 0.10–0.46 mmol/L, respectively. A smaller reference interval range was observed for TG in IDL, LDL and HDL subclass subfractions, for example, the reference interval range for TG in large HDL was 0.01–0.05 mmol/L.

**Table 2.** Reference interval range of 14 TG-subfractions (*n* = 9073).

TG subfraction (mmol/L)	2.50%	97.50%
Extremely large VLDL	0.01	0.06
Very large VLDL	<0.01	0.13
Large VLDL	0.01	0.42
Medium VLDL	0.08	0.67
Small VLDL	0.10	0.46
Very small VLDL	0.06	0.20
IDL	0.07	0.20
Large LDL	0.06	0.17
Medium LDL	0.02	0.08
Small LDL	0.01	0.05
Very large HDL	<0.01	0.03
Large HDL	0.01	0.05
Medium HDL	0.02	0.08
Small HDL	0.03	0.08

VLDL: very-low density lipoprotein; IDL: intermediate-density lipoprotein; LDL: low-density lipoprotein; HDL: high-density lipoprotein; TG: triglyceride.

### Subgroup reference interval ranges

Age and sex stratified reference interval ranges for 14 TG metabolites are presented in Supplemental Table 2. Reference interval ranges (2.5th–97.5th percentile) were comparable between male and female participants across metabolites. For example, among male participants aged <55 years, the large VLDL reference interval was in the range of 0.01–0.24 mmol/L, and for female participants of the same age band, the reference interval range was 0.02–0.03 mmol/L.

**Figure 2.**
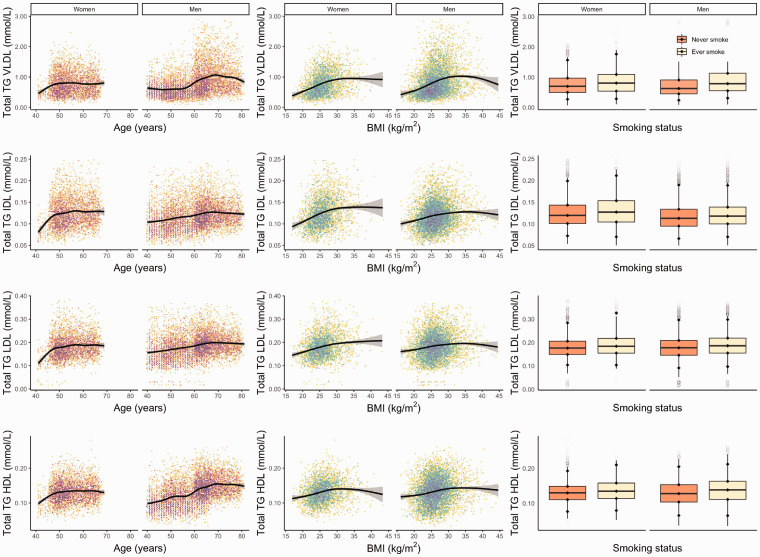
Distribution of TG concentration in VLDL, IDL, LDL and HDL subclass by age (left panel), body mass index (centre panel) and smoking status (right panel). N.b. Slope indicates a GAM estimate with 95% confidence interval. Tile colours represent the number of observations, with purple coloured tiles indicating a higher density. Smoking distribution (right panel) is based on data from BRHS, SABRE and WHII studies. All were significant at *P* value threshold for <0.00. VLDL: very-low density lipoprotein; IDL: intermediate-density lipoprotein; LDL: low-density lipoprotein; HDL: high-density lipoprotein; BRHS: British Regional Heart Study; SABRE: the Southall and Brent Revisited Study; WHII: the Whitehall II Study.Note. Please refer to the online version of the article to view the figure in colour.

Figure 2 shows GAM curves and density distribution for the sum of VLDL, IDL, LDL and HDL subclass subfractions for age and BMI, and box plot for TG distribution by smoking status. Among male participants, TG concentration increased with age, with the most prominent age differences observed in the HDL subclass (Figure 2, left panel). By comparison, TG concentration differences were not as noticeable for female participants for whom concentrations were comparable for the VLDL, IDL, LDL and HDL subclasses by age. For both men and women, TG subfraction concentration increased with increasing BMI for the VLDL, IDL, LDL and HDL subclasses. Ever smokers had higher mean TG subfraction concentrations across all subclasses as compared to never smokers.

In the disease subgroups that were removed from all previous analyses reported above, the reference interval ranges for 14 subfractions were comparable between those with CVD (N = 2719 and those with T2DM (N = 1325). In general, the largest variation in reference interval range across the subfractions between these subgroups was observed in the VLDL subclass (Supplemental Table 3). For example, the 2.5th to 97.5th reference interval range for TG in medium VLDL in CVD: 0.09, 0.79 mmol/L and in T2DM: 0.08, 0.84 mmol/L.

The reference interval ranges across 14 TG subfractions were comparable in TG measured in the fasting and non-fasting state and were higher in the group with clinical chemistry measured total TG greater than 1.7 mmol/L as compared to less than 1.7 mmol/L, see (Supplemental Table 3).

## Discussion

This study provides reference interval ranges for TG in 14 lipoprotein subfraction metabolites as measured by NMR spectroscopy based on a sample of UK adults. There was agreement in the distribution of TG subfraction concentrations between ethnicities. TG concentrations for male and female participants increase with increasing age and BMI, are higher among ever smokers and in those with CVD and T2DM as compared to disease-free subjects, and in individuals with total TG concentrations greater than 1.7 mmol/L.

Lipid reference interval ranges are derived using clinical chemistry measurement of blood samples from a reference population and are necessary to enable clinicians to apply analytical data in healthcare delivery. For example, clinical chemistry estimates of LDL-C are measured in individuals and evaluated against an interval range to inform lifestyle or therapeutic intervention for CHD prevention. The role of TG in CHD risk is less clear. Meta-analysis from prospective observational studies has demonstrated higher concentrations of clinical chemistry measured total TG are associated with higher risk of CHD, but effect estimates attenuate to the null after adjustment for HDL-C.^17^ On the other hand, Mendelian randomization studies support a potential causal association for TG.^18^ The association of the major blood lipid fractions (LDL-C, TG and HDL-C) with CHD is seen across the whole of the concentration range, with no threshold value and we expect the same to be true of TG-containing lipoprotein subfractions. The reference ranges we report should not therefore be taken to imply that individuals whose measurements lie within these ranges are free of CHD risk. Rather we simply report the observed values in general UK populations.

NMR methodology offers the potential for more granular quantification of TG in different lipoproteins that would otherwise be unavailable using conventional approaches, enabling a more detailed investigation of TG-containing lipoprotein subfractions in relation to CHD risk and prognosis. Evidence from studies using this approach suggest CHD risk may be divergent depending on the type of lipoprotein subfraction. Two recent studies report observations of TG in VLDL subfractions may be more atherogenic and associated with a higher risk of CHD compared to TG in the IDL, LDL and HDL subclass subfractions.^10^,^11^

This study evaluates the concentration distribution and range of TG in 14 subfractions and includes data from multiple UK population cohorts and from male and female participants from a range of age groups and ethnicities including European, South Asian and African-Caribbean ancestry. We compare total TG measured using clinical chemistry and the sum of NMR TG across the 14 subfractions. Discrepancies between clinical chemistry methods and NMR measured total TG have been reported previously.^10^,^17^ In one such study, Balling et al.^17^ suggest differences in analytical calibration from measurement of TGs between the two methods may lead to measurement differences, with NMR quantification deemed as the more accurate method.^18^ TG concentrations are variable and, in addition to age, sex and ethnicity, can depend on factors such as food intake, fasting/non-fasting state, CVD and metabolic disorders such as T2DM.^19^ Due to the relatively large sample sizes available, we observed a significant difference between the fasting and non-fasting distribution of subfractions. This significant difference did not prove relevant for determining the reference interval ranges, which were comparable to two decimal points. Moreover, it is postulated that the non-fasting state predominates the 24-h cycle due to varying food intake patterns, and mean changes of +0.3 mmol/L from baseline TG measures do not translate to clinically significant differences. Higher TG concentrations are observed in postmenopausal vs. premenopausal female.^20^ We report comparable TG concentrations across age groups; however, it is possible that a greater difference in TG concentration may be observed in a larger sample of women aged >65 than are included here. Due to the small numbers of current smokers in the available data across the contributing cohorts, we stratified by ever and never smoking status, instead of the more informative ‘never’, ‘ex and ‘current’ smokers. We exclude participants with current CVD or T2DM in the main analyses; however, it is possible that the TG concentrations in the study population were altered by other diseases or by lipid-lowering medication, which we were not able to account for in this study. It is likely TG monitoring is likely to occur in individuals at risk of, or with current CVD. Therefore, we provide additional reference intervals in participants with CVD and T2D.

We establish reference interval ranges of 14 TG-containing lipoprotein subfractions for male and female by age, BMI, smoking status, CVD, T2DM and stratified by clinical chemistry measured total TG and fasting status for population-based cohorts from the UK population. Further studies would be needed to assess if the reference intervals presented here could be extended to a non-UK population and if the risks associated with the reference intervals identify a threshold within these ranges to inform CVD risk in a clinical setting. By doing so, the reference interval ranges may help to set realistic targets and guide research interests, contributing to the development of effective targeted TG-lowering therapies, aimed at, for example VLDL subfractions which may be the most atherogenic.^10^ NMR lipoprotein particle number and size have been assessed in relation to CHD; however, this study specifically presents reference range intervals for TG within the 14 subfractions.^21^ Further investigations would be needed to compare subfraction lipid composition, particle number concentration and size.

Metabolomics is becoming integrated with genomics to contribute to a better understanding of disease aetiologies and disease risk.^22^ It is likely that quantitative metabolomics will be incorporated into large biobanks, which would extend the relevance of sample collection and encourage the life-long assessment of metabolic health.^6^ TG subfraction reference interval ranges may help complement current routine clinical chemistry measures of lipids and become an integral tool in targeted patient management and improved disease risk prediction and prevention.

## Conclusion

This study is the first to establish reference interval ranges for 14 TG-containing lipoprotein subfraction metabolites, measured using the Nightingale NMR platform for men and women in a UK population. NMR measures of lipoproteins may provide insights into biological processes compared to clinical chemistry measures of TG and lead to greater insights for the role of TG in CVD, emphasizing the importance of appropriate reference interval ranges for future clinical decision making.

## Supplemental Material

sj-pdf-1-acb-10.1177_0004563220961753 - Supplemental material for Establishing reference intervals for triglyceride-containing lipoprotein subfraction metabolites measured using nuclear magnetic resonance spectroscopy in a UK population Click here for additional data file. Supplemental material, sj-pdf-1-acb-10.1177_0004563220961753 for Establishing reference intervals for triglyceride-containing lipoprotein subfraction metabolites measured using nuclear magnetic resonance spectroscopy in a UK population by Roshni Joshi, Goya Wannamethee, Jorgen Engmann, Tom Gaunt, Deborah A Lawlor, Jackie Price, Olia Papacosta, Tina Shah, Therese Tillin, Peter Whincup, Nishi Chaturvedi 7 Mika Kivimaki, Diana Kuh, Meena Kumari, Alun D Hughes, Juan P Casas, Steve E Humphries, Aroon D Hingorani, A Floriaan Schmidt and on behalf of the UCLEB Consortium in Annals of Clinical Biochemistry
