# Supplementary material for: Establishing reference intervals for triglyceride-containing lipoprotein subfraction metabolites measured using nuclear magnetic resonance spectroscopy in a UK population
Source: Ann Clin Biochem. 2020 Oct 21;58(1):47–53. doi: 10.1177/0004563220961753 (PMC7791273; doi:10.1177/0004563220961753)
Supplement: sj-pdf-1-acb-10.1177_0004563220961753 - Supplemental material for Establishing reference intervals for triglyceride-containing lipoprotein subfraction metabolites measured using nuclear magnetic resonance spectroscopy in a UK population [file sj-pdf-1-acb-10.1177_0004563220961753.pdf]

Supplementary figures and tables

Supplementary figure 1 Scatter plot to show the sum of TG across 14 subfraction vs clinical chemistry measured total TG

Supplementary figure 2 Histograms to show the distribution of contributing UCLEB studies for 14 TG sub-fractions

Supplementary figure 3 Comparison of fasting vs non-fasting 14 TG sub-fraction measures from the SABRE cohort

N.b. Fasting and non-fasting samples N = 2260 (mmol/L); Kolmogorov-Smirnov test paired p-value

Supplementary table 1 Description of study populations

Supplementary table 2 Age and sex stratified reference interval ranges (2.5th, median 97.th percentile)

Supplementary table 3 CVD, T2DM, clinical chemistry measured total TG stratified reference interval ranges (2.5th, median, 97.5th percentile

Supplementary figure 1 Scatter plot to show the sum of TG across 14 subfraction vs clinical chemistry measured total TG

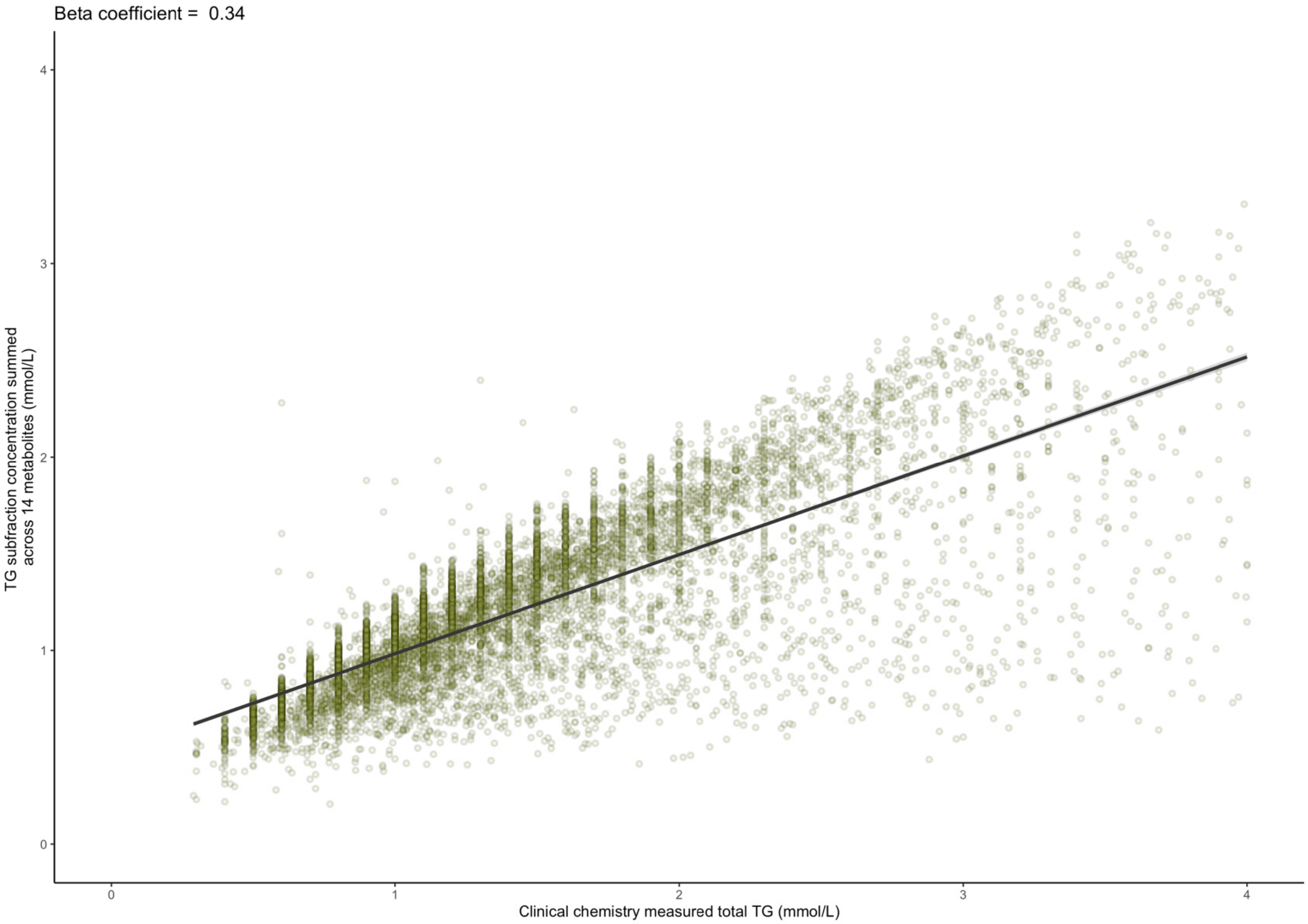

Supplementary figure 2 Histograms to show the distribution of contributing UCLEB studies for 14 TG sub-fractions

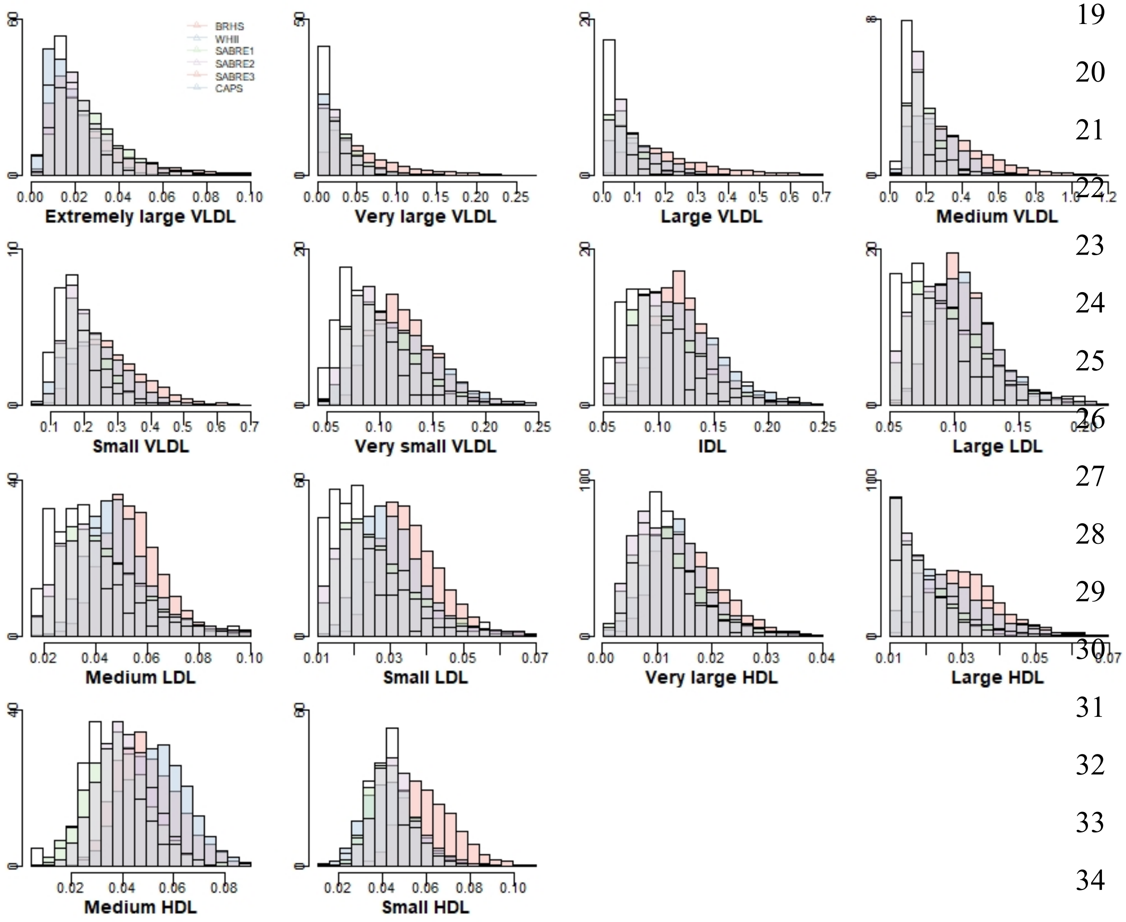

Supplementary figure 3 Comparison of fasting vs non-fasting 14 TG sub-fraction measures from the SABRE cohort

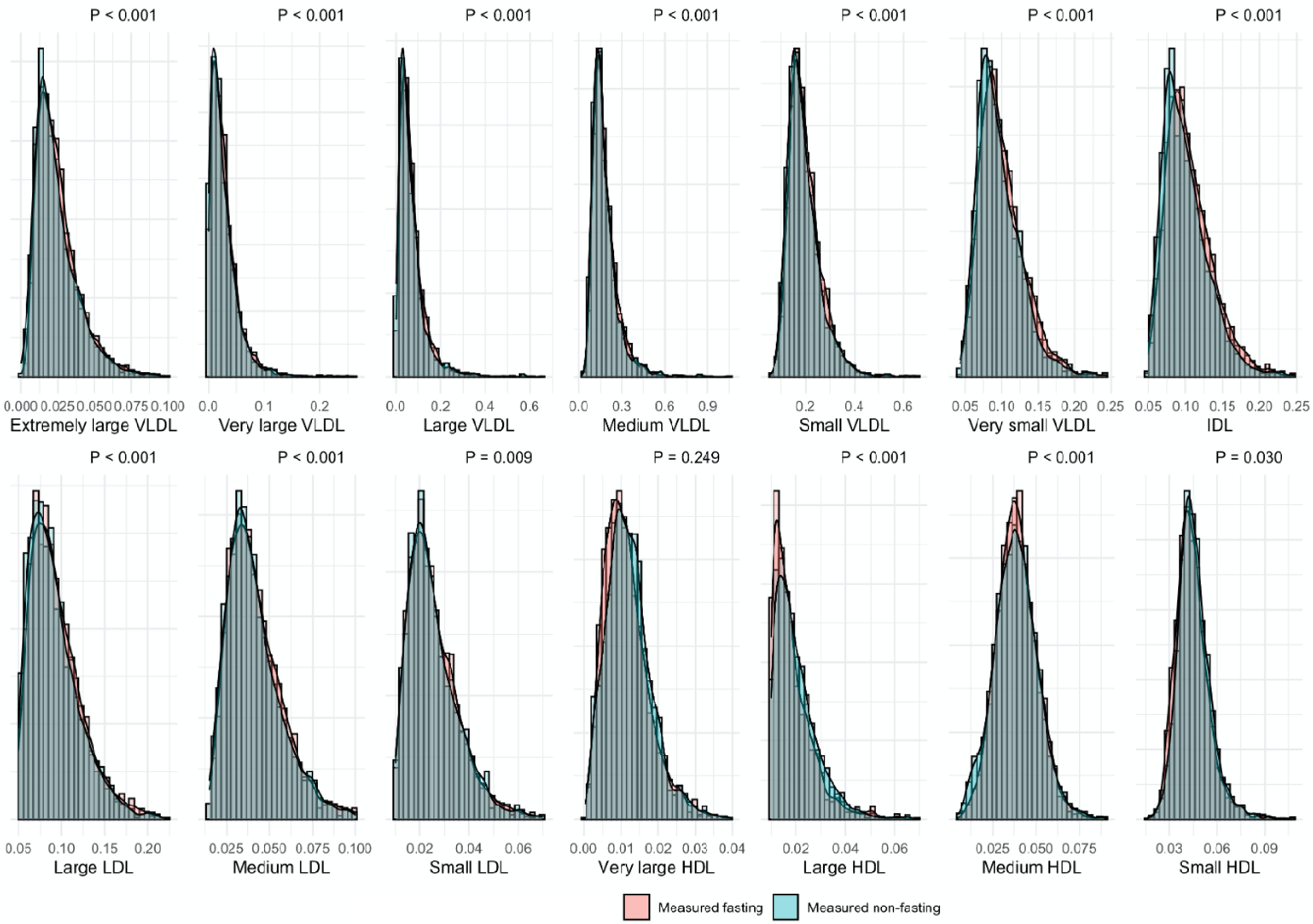

N.b. Fasting and non-fasting samples N = 2260 (mmol/L); Kolmogorov-Smirnov test paired p-value

Supplementary table 1 Description of study populations

	**SABRE2**<br>**N= 996**	**SABRE1**<br>**N= 1162**	**SABRE3**<br>**N= 102**	**WHII**<br>**N= 4042**	**BRHS**<br>**N= 2727**	**CAPS**<br>**N= 44**	**Total participant**<br>**sample N = 9,073**
Sample fasting status	**Fasting**	**Fasting**	**Fasting**	**Fasting and non-fasting**	**Fasting and non-fasting**	**Fasting**	
Age, years	51.1 (7.0)	53.2 (7.3)	53.3 (5.7)	55.5 (6.0)	68.7 (5.5)	62.3 (4.5)	58.1 (8.3)
Sex, male (%)	820 (82.3)	1002 (86.2)	97 (95.1)	1267 (26.8)	2727 (100)	44 (100)	5774 (63.6)
BMI, kg/m^2	26.1 (3.6)	26.1 (3.9)	26.8 (3.7)	26.1 (3.9)	26.9 (3.6)	27.7 (3.2)	26.2 (3.8)
Smoking, ever	207 (20.8)	808 (70.3)	39 (38.4)	515 (12.6)	1889 (69.2)	-	3458 (48.1)

Values are mean ± SD or %. BMI = Body mass index

SABRE: The Southall And Brent REvisited Study (2: South Asian, 1: Caucasian, 3: African-Caribbean ethnicities); WHII: the Whitehall II study; BRHS: British Regional Heart Study; BMI: body mass index; SBP: systolic blood pressure

Supplementary table 2 Age and sex stratified reference interval ranges (2.5th, median 97.th percentile)

	Men								
	<55 (n = 1825)			55 to 65 (n = 1910)			>65 (n = 1921)		
	2.50%	median	97.50%	2.50%	median	97.50%	2.50%	median	97.50%
Extremely large VLDL	<0.01	0.02	0.06	0.01	0.02	0.07	0.01	0.02	0.07
Very large VLDL	<0.01	0.02	0.09	<0.01	0.03	0.15	0.01	0.04	0.16
Large VLDL	0.01	0.06	0.24	0.01	0.10	0.48	0.04	0.16	0.52
Medium VLDL	0.07	0.16	0.43	0.07	0.24	0.73	0.12	0.32	0.82
Small VLDL	0.09	0.18	0.37	0.10	0.23	0.48	0.13	0.26	0.51
Very small VLDL	0.05	0.01	0.18	0.06	0.11	0.19	0.07	0.12	0.19
IDL	0.06	0.11	0.19	0.07	0.12	0.19	0.08	0.12	0.19
Large LDL	0.05	0.09	0.17	0.06	0.10	0.17	0.07	0.11	0.17
Medium LDL	0.02	0.04	0.08	0.03	0.05	0.08	0.03	0.05	0.08
Small LDL	0.01	0.03	0.05	0.01	0.03	0.05	0.02	0.03	0.05
Very large HDL	<0.01	0.01	0.03	<0.01	0.01	0.03	0.01	0.02	0.03
Large HDL	0.01	0.02	0.05	0.01	0.03	0.05	0.02	0.03	0.06
Medium HDL	0.02	0.04	0.07	0.02	0.05	0.08	0.03	0.05	0.08
Small HDL	0.02	0.04	0.07	0.03	0.05	0.08	0.04	0.06	0.09
	Women								
	<55 (n = 1839)			>55 to <65 (n =1243)			>65 (n =204)		
	2.50%	median	97.50%	2.50%	median	97.50%	2.50%	median	97.50%
Extremely large VLDL	0.01	0.02	0.05	0.01	0.02	0.04	0.01	0.02	0.04
Very large VLDL	<0.01	0.02	0.09	<0.01	0.02	0.08	<0.01	0.02	0.08
Large VLDL	0.02	0.09	0.30	0.02	0.09	0.29	0.03	0.10	0.29

Medium VLDL	0.08	0.23	0.54	0.08	0.24	0.54	0.10	0.25	0.54
Small VLDL	0.10	0.23	0.44	0.10	0.23	0.43	0.12	0.24	0.42
Very small VLDL	0.06	0.11	0.20	0.06	0.12	0.20	0.07	0.12	0.19
IDL	0.07	0.12	0.20	0.08	0.12	0.20	0.08	0.12	0.20
Large LDL	0.06	0.10	0.17	0.07	0.11	0.16	0.07	0.11	0.17
Medium LDL	0.02	0.04	0.08	0.03	0.05	0.08	0.03	0.05	0.07
Small LDL	0.01	0.03	0.05	0.02	0.03	0.05	0.02	0.03	0.05
Very large HDL	<0.01	0.01	0.03	<0.01	0.01	0.03	0.01	0.01	0.02
Large HDL	0.01	0.02	0.05	0.01	0.02	0.05	0.01	0.02	0.05
Medium HDL	0.03	0.05	0.08	0.03	0.05	0.08	0.03	0.05	0.07
Small HDL	0.03	0.04	0.07	0.02	0.04	0.07	0.03	0.04	0.06

Supplementary table 2 Reference interval range of 14 TG sub-fractions stratified by CVD, T2DM, clinical chemistry measured total TG greater and less than 1.7 mmol/L and measured in the fasting and non-fasting state.

CVD (n = 2719)			
	2.50%	median	97.50%
Extremely large VLDL	0.01	0.02	0.07
Very large VLDL	<0.01	0.04	0.17
Large VLDL	0.02	0.13	0.53
Medium VLDL	0.09	0.29	0.79
Small VLDL	0.12	0.25	0.51
Very small VLDL	0.07	0.12	0.20
IDL	0.07	0.12	0.20
Large LDL	0.06	0.10	0.17
Medium LDL	0.03	0.05	0.08
Small LDL	0.02	0.03	0.05
Very large HDL	<0.01	0.01	0.03
Large HDL	0.01	0.03	0.05
Medium HDL	0.03	0.05	0.08
Small HDL	0.03	0.05	0.09
T2DM (n = 1325)			
	2.50%	median	97.50%
Extremely large VLDL	0.01	0.02	0.08
Very large VLDL	<0.01	0.04	0.18
Large VLDL	0.02	0.13	0.55
Medium VLDL	0.08	0.27	0.84
Small VLDL	0.12	0.25	0.52
Very small VLDL	0.06	0.12	0.21
IDL	0.07	0.12	0.21
Large LDL	0.06	0.11	0.18
Medium LDL	0.02	0.05	0.09
Small LDL	0.01	0.03	0.06
Very large HDL	<0.01	0.01	0.03
Large HDL	0.01	0.02	0.05
Medium HDL	0.03	0.05	0.08
Small HDL	0.03	0.05	0.09
Clinical chemistry measured total TG < 1.7 mmol/L (n = 6076)			
Extremely large VLDL	<0.01	0.01	0.03
Very large VLDL	<0.01	0.02	0.05

Large VLDL	0.01	0.07	0.19
Medium VLDL	0.07	0.19	0.37
Small VLDL	0.09	0.19	0.32
Very small VLDL	0.06	0.10	0.16
IDL	0.07	0.11	0.17
Large LDL	0.06	0.10	0.15
Medium LDL	0.02	0.04	0.07
Small LDL	0.01	0.03	0.04
Very large HDL	<0.01	0.01	0.02
Large HDL	0.01	0.03	0.05
Medium HDL	0.02	0.04	0.07
Small HDL	0.02	0.04	0.06
Clinical chemistry measured total TG > 1.7 mmol/L (n = 2860)			
Extremely large VLDL	0.01	0.03	0.08
Very large VLDL	0.01	0.06	0.17
Large VLDL	0.03	0.22	0.53
Medium VLDL	0.12	0.41	0.82
Small VLDL	0.15	0.32	0.53
Very small VLDL	0.08	0.14	0.22
IDL	0.08	0.14	0.21
Large LDL	0.07	0.12	0.18
Medium LDL	0.03	0.06	0.09
Small LDL	0.02	0.04	0.06
Very large HDL	<0.01	0.02	0.03
Large HDL	0.01	0.03	0.05
Medium HDL	0.03	0.06	0.08
Small HDL	0.04	0.06	0.09
Fasting state (N= 2273)			
Extremely large VLDL	0.01	0.02	0.06
Very large VLDL	<0.01	0.02	0.09
Large VLDL	0.01	0.06	0.24
Medium VLDL	0.08	0.16	0.42

Small VLDL	0.11	0.19	0.36
Very small VLDL	0.06	0.09	0.17
IDL	0.06	0.1.0	0.18
Large LDL	0.05	0.09	0.17
Medium LDL	0.02	0.04	0.08
Small LDL	0.01	0.02	0.05
Very large HDL	<0.01	0.01	0.03
Large HDL	0.01	0.02	0.04
Medium HDL	0.02	0.04	0.06
Small HDL	0.03	0.04	0.07
Non-fasting state (N= 2273)			
Extremely large VLDL	0.01	0.02	0.06
Very large VLDL	<0.01	0.02	0.10
Large VLDL	0.01	0.05	0.25
Medium VLDL	0.07	0.16	0.44
Small VLDL	0.10	0.18	0.36
Very small VLDL	0.06	0.09	0.17
IDL	0.06	0.09	0.17
Large LDL	0.05	0.09	0.16
Medium LDL	0.02	0.04	0.08
Small LDL	0.01	0.02	0.05
Very large HDL	<0.01	0.01	0.03
Large HDL	0.01	0.02	0.04
Medium HDL	0.01	0.04	0.06
Small HDL	0.03	0.04	0.07
